# The African Network for Improved Diagnostics, Epidemiology and Management of common infectious Agents

**DOI:** 10.1186/s12879-021-06238-w

**Published:** 2021-06-07

**Authors:** Grit Schubert, Vincent Achi, Steve Ahuka, Essia Belarbi, Ouattara Bourhaima, Tim Eckmanns, Siobhan Johnstone, Firmin Kabore, Ouffoue Kra, Adriano Mendes, Abdoul-Salam Ouedraogo, Armel Poda, Arsène Satouro Some, Sara Tomczyk, Emmanuel Couacy-Hymann, Jean-Marie Kayembe, Nicolas Meda, Jean-Jacques Muyembe Tamfum, Soumeya Ouangraoua, Nicola Page, Marietjie Venter, Fabian H. Leendertz, Chantal Akoua-Koffi, the ANDEMIA consortium, Vincent Assé Kouadio, Aude Aka-Tano, Adoulaye Diarrassouba, Etilé Anoh, Adjaratou Traoré, Fidèle Touré Sounan, Safiatou Karidioula, Gabriel Mbunsu Kizito, Benilde Bepouka Izizag, Nicole Mpwekela, Benoit Kabengele, Nicole Alama, Olivier Tshiani, Eddy Kinganda Lusamaki, Baby Muyembe, Naomie Mitongo, John Manienga, Franck Lionzo, Alliance Mbandu, Sheila Makiala, Muna Abu Sin, Karin Gröschner, Susanne Köhler, Sandra Niendorf, Kathrin Nowak, Paul Pitzinger, Andreas Sachse, Ann Christin Vietor, Juno Thomas, Sibongile Walaza, Linda de Gouvea, Claire von Mollendorf, Vanessa Quan, Karen Keddy, Anthony Smith, Ntsieni Ramalwa, Theunis Avenant, Nicolette du Plessis, Kgothatso Menu, Marthi Pretorius, Caitlyn McIntyre, Elise Bonnet, Rebecca Jeal

**Affiliations:** 1 Robert Koch-Institute, Berlin, Germany; 2 Centre Hospitalier Universitaire Bouaké, Bouaké, Côte d’Ivoire; 3 Université Alassane Ouattara de Bouaké, Bouaké, Côte d’Ivoire; 4 Institut National de la Recherche Biomédicale, Kinshasa, Democratic Republic of the Congo; 5 National Institute for Communicable Diseases, Johannesburg, Republic of South Africa; 6 Centre Muraz, Bobo-Dioulasso, Burkina Faso; 7 University of Pretoria, Pretoria, Republic of South Africa; 8 Centre Hospitalier Universitaire Sourô Sanou de Bobo-Dioulasso, Bobo-Dioulasso, Burkina Faso; 9 Laboratoire National d’Appui au Développement Agricole / Laboratoire Central de Pathologie Animale, Bingerville, Côte d’Ivoire; 10 Hôpital Universitaire/Université de Kinshasa, Kinshasa, Democratic Republic of the Congo

**Keywords:** Acute respiratory tract infections, Acute gastrointestinal infections, Acute febrile disease of unknown cause, Sentinel surveillance, Sub-Saharan Africa, Aetiologies, Outbreak detection, Antimicrobial resistance, SARS-CoV-2, COVID-19

## Abstract

**Background:**

In sub-Saharan Africa, acute respiratory infections (ARI), acute gastrointestinal infections (GI) and acute febrile disease of unknown cause (AFDUC) have a large disease burden, especially among children, while respective aetiologies often remain unresolved. The need for robust infectious disease surveillance to detect emerging pathogens along with common human pathogens has been highlighted by the ongoing novel coronavirus disease 2019 (COVID-19) pandemic. The African Network for Improved Diagnostics, Epidemiology and Management of Common Infectious Agents (ANDEMIA) is a sentinel surveillance study on the aetiology and clinical characteristics of ARI, GI and AFDUC in sub-Saharan Africa.

**Methods:**

ANDEMIA includes 12 urban and rural health care facilities in four African countries (Côte d’Ivoire, Burkina Faso, Democratic Republic of the Congo and Republic of South Africa). It was piloted in 2018 in Côte d’Ivoire and the initial phase will run from 2019 to 2021. Case definitions for ARI, GI and AFDUC were established, as well as syndrome-specific sampling algorithms including the collection of blood, naso- and oropharyngeal swabs and stool. Samples are tested using comprehensive diagnostic protocols, ranging from classic bacteriology and antimicrobial resistance screening to multiplex real-time polymerase chain reaction (PCR) systems and High Throughput Sequencing. In March 2020, PCR testing for severe acute respiratory syndrome coronavirus 2 (SARS-CoV-2) and analysis of full genomic information was included in the study. Standardised questionnaires collect relevant clinical, demographic, socio-economic and behavioural data for epidemiologic analyses. Controls are enrolled over a 12-month period for a nested case-control study. Data will be assessed descriptively and aetiologies will be evaluated using a latent class analysis among cases. Among cases and controls, an integrated analytic approach using logistic regression and Bayesian estimation will be employed to improve the assessment of aetiology and associated risk factors.

**Discussion:**

ANDEMIA aims to expand our understanding of ARI, GI and AFDUC aetiologies in sub-Saharan Africa using a comprehensive laboratory diagnostics strategy. It will foster early detection of emerging threats and continued monitoring of important common pathogens. The network collaboration will be strengthened and site diagnostic capacities will be reinforced to improve quality management and patient care.

## Background

Pneumonia, diarrhoea and fevers persist as frequent reasons for seeking healthcare in low- and middle-income countries, particularly among children [1–5]. Sub-Saharan Africa (SSA) bears a disproportionately high burden of morbidity and mortality due to such common infections [6–8]. While health interventions like the introduction of the pneumococcal and *Haemophilus influenzae* vaccines [9], or improvements of sanitation have contributed to overall declines in under-five mortality attributable to acute respiratory infections (ARI), gastrointestinal infections (GI) and acute febrile disease of unknown cause (AFDUC) since 1990 [10, 11], they remain among the top five causes of death in sub-Saharan Africa [8]. The Global Enteric Multicenter Study (GEMS) found that the six most frequent pathogens causing GI in children less than 5 years in African and Asian low- and middle-income countries were *Shigella* spp., rotavirus, adenovirus, enterotoxigenic *Escherichia coli* (ETEC), Cryptosporidium spp. and Campylobacter spp. [12]. The Pneumonia Etiology Research for Child Health (PERCH) study also found that a small set of pathogens accounted for most cases of severe pneumonia in children requiring hospital admission in sub-Saharan Africa and Asia, dominated by *Streptococcus pneumoniae*, respiratory syncytial virus, human metapneumovirus and rhinovirus [13].

Undifferentiated non-malarial fever makes up a significant proportion of childhood disease burden in Africa and Asia [14, 15]. AFDUC is often investigated in single pathogen studies, e.g. on typhoid fever [16]. It is less frequently assessed at the syndrome level, as it can have a multitude of infectious causes and therefore requires broad diagnostics [1, 17]. A recent systematic review identified *Staphylococcus aureus*, non-typhoidal *Salmonella* and *Escherichia coli* to be the most commonly reported bacterial causes, and arboviruses to be among the most common viral causes of AFDUC in SSA [5]. This review highlighted the lack of data from rural areas, which tends to be neglected by surveillance efforts. In addition, febrile illness often remains undiagnosed due to the lack of broad routine laboratory testing. For example, pathogens other than *Plasmodium* spp. were found to cause febrile disease in children less than 5 years in nearly half of the investigated cases in Burkina Faso, while in 24% of the cases, no causative agent was detected [18]. Recently, the Febrile Illness Evaluation in a Broad Range of Endemicities (FIEBRE) study was established to investigate causes of febrile illness in three SSA countries as well as two Asian countries [19].

The aforementioned evidence underscores the complexity and interrelatedness of ARI, GI and AFDUC, where symptoms and causative agents often overlap. ARI and GI are associated with fevers, but these patients are often only tested for common respiratory or gastrointestinal pathogens, leaving a proportion of aetiologies unresolved. While large-scale surveillance programs have investigated each syndrome on its own (e.g. the PERCH study among those with severe pneumonia [13], the GEMS study among those with GI [20] and the FIEBRE study among those with febrile illness but excluding ARI and GI cases [19]), simultaneous surveillance of all three highly related disease syndromes, across ecosystems and demographic settings, is still lacking to date. These syndromes are often treated empirically due to limited access to broad high-quality laboratory diagnostics, which hinders informed treatment decisions and targeted health interventions. A comprehensive surveillance program could help address important knowledge gaps, particularly in the understanding of AFDUC or of other threats such as antimicrobial resistance (AMR). Furthermore, it can function as an early warning system for infectious disease outbreaks, such as the COVID-19 pandemic [21]. By including acute respiratory infection, the system can provide a platform for monitoring and researching the disease dynamics of SARS-CoV-2, which is currently spreading in SSA [22].

The African Network for Improved Diagnostics, Epidemiology and Management of Common Infectious Agents (ANDEMIA) is a transnational sentinel surveillance study on the aetiology and clinical characteristics of ARI, GI and AFDUC in SSA. Sites are established in Burkina Faso (BF), Côte d’Ivoire (CIV), the Democratic Republic of the Congo (DRC) and the Republic of South Africa (RSA), countries which carry a significant burden of ARI, GI and AFDUC [8]. In an effort to fully elucidate the extent of the disease burden, ANDEMIA is inclusive of all age groups and will not limit its research to easy-access hospital sites but also include rural regions. Especially in regions with high biodiversity and close human-animal contact, zoonotic pathogens are at the source of emerging infections, which may be related to causes of AFDUC, ARI and GI as well [23]. ANDEMIA aims to improve clinical microbiological practice, implement advanced molecular diagnostics, and collect broad surveillance data on ARI, GI and AFDUC at the selected sites.

## Methods and setting

### Study aims

The overall aims of the ANDEMIA surveillance study are to build capacities and strengthen the network collaboration to enable the enhanced detection, control, treatment and prevention of ARI (including COVID-19), GI and AFDUC as well as the spread of AMR in SSA. Specifically, the study will:

1. identify the aetiologies found in cases of ARI, GI, and AFDUC syndromes using large pathogen test panels;
2. assess the associated clinical, demographic and socio-economic characteristics and behavioural factors;
3. determine the antibiotic resistance profiles of relevant bacteria; and.
4. investigate the molecular epidemiology of relevant etiological agents.

ANDEMIA aims to use surveillance data for developing targeted interventions, assessing their effectiveness and informing further in-depth investigations. Our assumption is that locally-adapted interventions based on broad integrated clinical, epidemiological and laboratory surveillance data will more effectively improve patient outcomes and disease prevention efforts than vertical disease programs. Moreover, we will also put focus on a ‘One Health’ approach to further explore pathogen emergence and the intersection of human and animal health including aspects such as antibiotic use and exposure to zoonotic pathogens. Since the surveillance study was established before the COVID-19 pandemic emerged, ANDEMIA also presents a valuable opportunity to compare data from before and throughout the course of the pandemic to detect SARS-CoV-2 trends and identify risk factors for infection, disease severity, co-infections, as well as the molecular dynamics of the virus through High Throughput Sequencing.

### Study design and sentinel sites

**Fig. 1:**
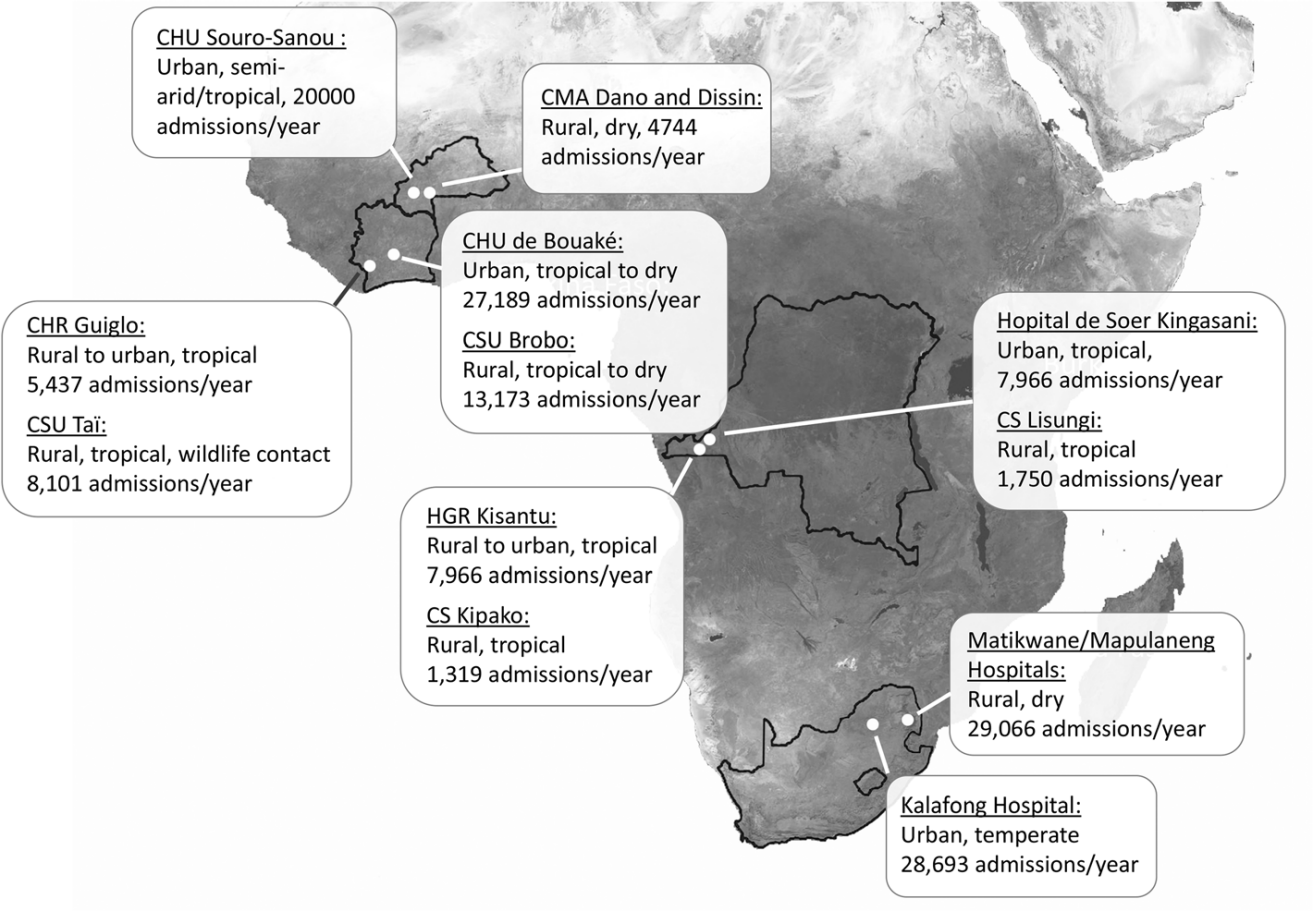
ANDEMIA study sites. Legend: CHR, Centre Hospitalier Regional; CHU, Centre Hospitalier et Universitaire; CS, Centre de Santé; CSU, Centre de Santé Urbain; CMA, Centre Médical avec Antenne chirurgicale; HGR, Hospital General Regional. Map taken from NASA, Public domain

**Fig. 2:**
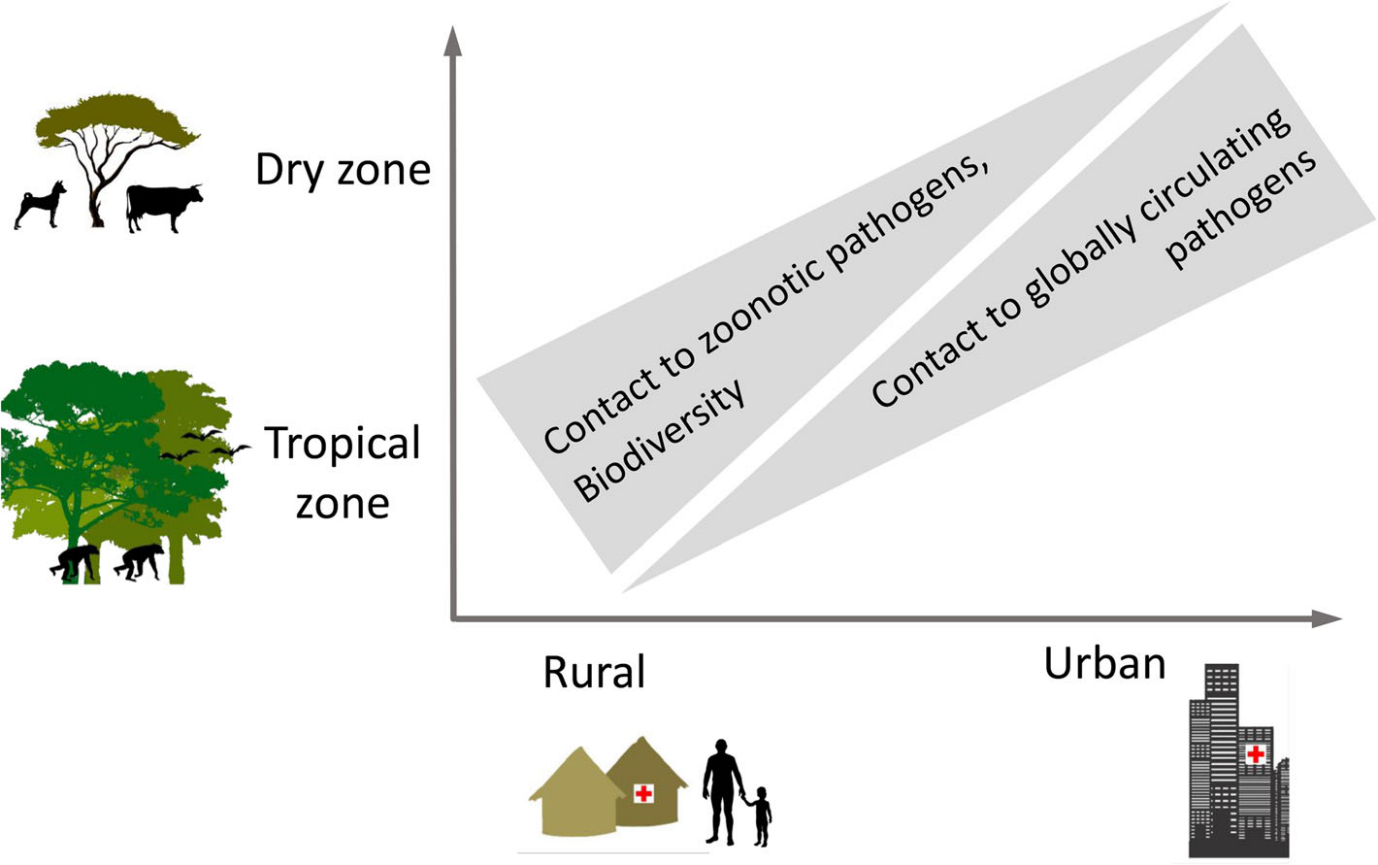
ANDEMIA study design. Sentinel hospital sites are located along gradients from rural to urban, as well as dry to tropical climate to account for the various effects that demography, climate and biodiversity may have on local pathogen compositions. Image edited with Adobe Photoshop CS6

ANDEMIA is a prospective syndromic hospital-based sentinel surveillance study at 12 hospital sentinel sites (Fig. 1) in the African countries BF, CIV, DRC and RSA. After a pilot phase in 2018 at CIV, it will initially run from 2019 to 2021 and then be evaluated. CIV and DRC have two urban and two rural sentinel sites whereas RSA and BF have one urban and one rural sentinel site each, which are situated in different climatic and biodiversity settings. Urban sites are characterized by higher population density and mobility and access to acute care hospitals. Rural sites are characterized by lower population density and mobility, where patients attend smaller health centres and have closer contact to wildlife and/or livestock. The rationale behind this surveillance study design is that exposure to e.g. zoonotic pathogens or globally circulating microorganisms including antimicrobial resistant pathogens is expected to vary between the sentinel sites (Fig. 2). Including a range of sites will allow us to better investigate the effect of climate, biodiversity and demography on human health in a ‘One Health’ framework.

Sentinel surveillance is coordinated by each country’s partner institutions, i.e. the Centre Hospitalier Universitaire (CHU) Bouaké and the Laboratoire National d’Appui au Développement Agricole / Laboratoire Central de Pathologie Animale (LANADA) in CIV, the Centre Muraz and the CHU de Sourô Sanou in BF, the Institut National de la Recherche Biomédicale (INRB) and the Hôpital Universitaire/Université de Kinshasa in DRC and the National Institute for Communicable Diseases (NICD) and University of Pretoria in RSA. Technical support according to needs identified by partners and advanced epidemiological and laboratory analyses are provided by the Robert Koch-Institute in Germany. Figure 1 displays demographic characteristics of all sites. The program is coordinated by the CHU Bouaké, CIV, and the Robert Koch-Institute (RKI, Germany).

### Study participants and sample size

**Fig. 3:**
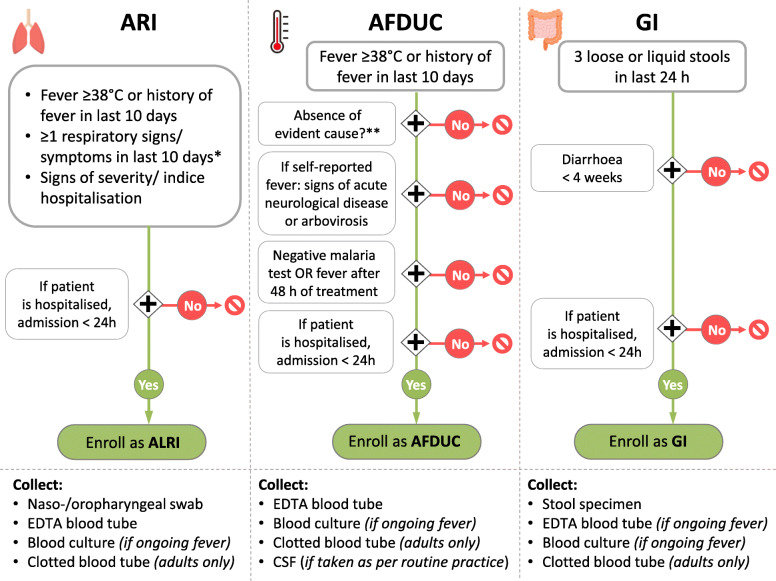
Case definitions decision tree. Legend: *respiratory symptoms are cough, expectoration, dyspnoea, pulmonary consolidation, chest pain; **other than acute neurological disease or arbovirosis, evident causes are soft tissue infection (including dental abscess, severe gum or mouth infection) or muscle infection, severe surgical condition or surgical abdomen, kidney or urinary tract infection, HIV Opportunistic Diseases (WHO, 2010b) and Acute Retroviral Syndrome. ARI, acute respiratory infection; AFDUC, acute febrile disease of unknown cause; GI, acute gastrointestinal infection

The study population includes patients of all ages that present to the sentinel sites, who meet the ARI, GI or AFDUC case definitions and provide informed consent. Case definitions was based on published surveillance standards [ARI: [24], GI: [25], AFDUC: [26, 27]] and network expert consensus (Fig. 3). Patients are enrolled either as ARI (including suspected COVID-19 disease), GI or AFDUC cases alone, or as cases with both ARI and GI.

Exclusion criteria include:

1. for AFDUC patients: being malaria-positive and not yet 48 h under malaria therapy; therefore, malaria testing is conducted (rapid test or blood smear) and data are recorded for all AFDUC patients presenting with a fever ≥38 °C;
2. new-borns who have not been discharged following delivery; and.
3. patients transferred directly to intensive care units without passing through the admission ward.

The planned sample size is 320 patients per syndrome per year per site. As such, the study is expected to provide sufficient power to detect aetiologies and pathogens of interest. The sample size calculation was based on a two-sided test, 5% significance level, 80% power, and an expected aetiology of 10% based on previous literature from respiratory disease surveillance in SSA (e.g. influenza virus) [28]. This target sample size may be more difficult to meet in rural sites if fewer patient admissions are seen. Sensitivity analyses will be run to evaluate the ongoing results and observed margins of error.

### Enrolment, informed consent and questionnaire

Patients meeting case definitions are identified by surveillance officers or medical staff trained in the ANDEMIA protocol upon presentation at the hospital. Surveillance officers discuss the study with the identified patients and provide them with a study information sheet explaining the objectives of the surveillance study, the benefits of participation and potential risks. If they agree to participate in the surveillance study, they are asked to sign standardised informed consent forms. Using a standardized structured case investigation form, surveillance officers collect clinical, demographic, socio-economic and behavioural data (Table 1). Further ethical considerations are detailed in the ‘Declarations’ section.

**Table 1:** Key data recorded from ANDEMIA patients

Category	Data collected
Demographic	Sex
	Age
Clinical	Physical examination data
	Specific symptoms related to ARI, AFDUC or GI
	Pre-existing conditions
	Malaria history
	Hospitalization history
	Human Immunodeficiency Virus (HIV) status
	Tuberculosis status
	Recent medication record
	Vaccination history
Socio-economic/behavioral factors	Education level
	Living conditions/water and sanitation
	Exposure to animals and animal products
	Travel history
One-month follow-up	Disease outcome (recovered/ongoing symptoms/deceased)

Legend: *ARI* acute respiratory infection; *AFDUC* acute febrile disease of unknown cause; *GI* acute gastrointestinal infection

### Sampling procedures

Specimens are collected right after questionnaire completion by study staff and respective sampling forms are completed. Whole blood specimens are collected in bacterial culture bottles as well as Ethylenediaminetetraacetic acid (EDTA) tubes. Clotted blood tubes for subsequent serological analyses are collected from adult febrile patients. Among ARI patients, naso- and oropharyngeal swabs are collected and stored in universal transport media (UTM). Among GI patients, stool specimens or rectal wipes or swabs are collected. In addition, an aliquot of cerebrospinal fluid (CSF) will be included if a respective specimen is requested for routine diagnostics by the treating physician.

### Nested case-control study

One set of healthy controls will also be enrolled as part of the surveillance study. The nested case-control study will enable the further investigation of aetiologies of active infection versus colonization and the association between infection outcomes and risk factors. Controls will receive the study information sheet and consent to study participation by signing a consent form. Each country is similarly expected to enrol 320 controls over a 12-month period in order to capture seasonal variation, and controls will be split to match frequency of case enrolment 1:1 at the sentinel sites. Controls will be frequency-matched by age group (infants < 1 year of age; young children 1–4 years of age; children 5–17 years of age; adults 18–44 years of age; older adults > 44 years of age).

Ongoing sensitivity analyses will assess the power to detect significant differences between proportions of cases and controls for which a specific pathogen is found. Inclusion criteria for controls are: 1) no symptoms of GI infection (including diarrhoea, vomiting), ARI (including cough, expectoration, pulmonary consolidation, chest pain, pneumonia, apnoea) or fever, rash, arthralgia, and signs or symptoms of neurological disease in the previous 3 weeks; and 2) providing informed consent. Exclusion criteria for controls are: 1) being a family member or caregiver of an ANDEMIA case; 2) admitted to the hospital for longer than 48 h; 3) being pregnant; 4) use of antibiotics in the past 24 h; and 5) previously enrolled as an ANDEMIA case or control. The case investigation form will be completed, and sampling as well as laboratory analyses will be equivalent to ANDEMIA patients with the exclusion of bacterial culturing (Table 2).

**Table 2:** Biological sampling and laboratory tests conducted in the ANDEMIA study

Biological samples	Study population	Laboratory testing
Whole blood	Febrile cases	Blood culture
	Cases/ healthy controls	Molecular detection of bacteria (FTD® Bacterial pneumonia CAP) for RTI cases
	Cases/ healthy controls	Molecular detection of viruses and bacteria (multiplex PCR-based macroarray assay) for AFDUC cases and RTI/GI cases without first-line pathogen detection
Naso−/ Oropharyngeal swabs	Cases/ healthy controls	Molecular detection of viruses and bacteria (FTD® respiratory pathogens 33)
	Cases	Molecular detection of SARS-CoV-2 (TIB Molbiol LightMix® SarbecoV E-gene and SARS-CoV-2 RdRP-gene) from March 2020 onwards, and retrospectively from December 2019 on
Stool	Cases	Stool culture
	Cases/ healthy controls	Molecular detection of viruses (FTD® viral gastroenteritis), bacteria (FTD® bacterial gastroenteritis) and parasites (FTD® stool parasites)

Legend: *ARI* acute respiratory infection; *GI* acute gastrointestinal infection; *AFDUC* acute febrile disease of unknown cause

### Laboratory testing and quality management

Main laboratory approaches are summarized in Table 2. After an initial culturing, positive whole blood and stool specimens are sub-cultured onto the appropriate selective and non-selective media and examined for the presence of respiratory, fever-associated and enteric pathogens. Microorganism identification and antimicrobial susceptibility testing will be performed at the partners’ laboratories following standardized protocols. Bacterial isolates are stored on site at − 80 °C for further molecular characterization.

Among ARI cases, a real-time multiplex polymerase chain reaction (PCR; FTD® Bacterial pneumonia CAP; Fast Track Diagnostics Luxembourg) is performed on nucleic-acid extracted from whole blood specimens to detect the following respiratory bacteria: *Streptococcus pneumoniae*, *Staphylococcus aureus*, *Haemophilus influenzae*, *Moraxella catarrhalis*, *Legionella pneumophila*/ *Legionella longbeachae*, *Mycoplasma pneumoniae* and *Chlamydia pneumoniae*. Naso- and oropharyngeal swabs are tested using multiplex real-time PCR testing for respiratory viruses and bacteria (FTD® respiratory pathogens 33; Fast Track Diagnostics Luxembourg). The assay targets influenza A, influenza B, influenza C, influenza A (H1N1) viruses, parainfluenza viruses 1, 2, 3 and 4, coronaviruses NL63, 229E, OC43 and HKU1, human metapneumoviruses A and B, rhinovirus, respiratory syncytial viruses A and B, adenovirus, enterovirus, parechovirus, bocavirus, *Pneumocystis jirovecii*, *Mycoplasma pneumoniae*, *Chlamydia pneumoniae*, *Streptococcus pneumoniae*, *Haemophilus influenzae* type B, *Staphylococcus aureus*, *Moraxella catarrhalis*, *Bordetella* spp. (except *Bordetella parapertussis*), *Klebsiella pneumoniae*, *Legionella pneumophila*/*Legionella longbeachae*, *Salmonella* spp. and *Haemophilus influenzae*. Real-time RT-PCR detection of SARS-CoV-2 (TIB Molbiol LightMix® SarbecoV E-gene and TIB Molbiol LightMix® SARS-CoV-2 RdRP) was implemented since March 2020 on extracted nucleic acids from respiratory specimens. Samples from November 2019 on were retrospectively tested as well [29].

Among GI patients, three real-time multiplex PCR systems are used on stool specimens to detect the following pathogens: the enteric viruses norovirus GI, norovirus II, human astrovirus, rotavirus, human adenovirus and sapovirus (FTD viral gastroenteritis, Fast Track Diagnostics Luxembourg), the enteric bacteria *Campylobacter coli*/*jejuni* /*lari*, *Clostridioides difficile*, *Escherichia coli* verotoxin positives, *Salmonella* spp., *Shigella* spp., enteroinvasive *Escherichia coli* and *Yersinia enterocolitica* (FTD bacterial gastroenteritis), and lastly the enteric parasites *Entamoeba histolytica*, *Cryptosporidium* spp. and *Giardia lamblia* (FTD stool parasites). Rotavirus-positive specimens will be genotyped using standardized methods and primers specific for G1, G2, G3, G4, G8, G9, G10, G12 and for P[4], P[6], P[8], P[9], P[10], P[11] and P[14] strains.

Among AFDUC patients, a multiplex PCR-based macroarray assay (*Fever Chip*) will be used on whole blood and CSF specimens for differential diagnosis of 30 pathogens associated with febrile disease, haemorrhagic fever disease and meningo-encephalitis including both common and less frequently encountered viruses, bacteria, parasites, and arboviruses of medical importance in SSA [30]. Pathogens included on the *Fever Chip* are Crimean-Congo haemorrhagic fever virus, Rift Valley Fever virus, West Nile virus, Chikungunya virus, Dengue virus, Sindbis virus, Rubella virus, Cytomegalovirus, Measles virus, Mumps virus, Herpes simplex 1 and 2 viruses, Varicella Zoster virus, Rabies virus, Epstein-Barr virus, JC virus, Enterovirus, Adenovirus, Flavivirus family, Hepatitis A and B viruses, *Rickettsia* spp., *Borrelia burgdorferi*/*garinii*, *Brucella* spp., *Coxiella burnetti*, *Leptospira* spp. *Mycobacterium tuberculosis*, *Ehrlichia* spp., *Neisseria meningitidis* and *Plasmodium* spp. The short viremia for certain arboviruses may necessitate the additional use of serological tests, e.g. for the Alphavirus and Flavivirus genera. In positive cases, neutralization tests will be used to differentiate between virus species in the Alphavirus (Sindbis virus; Chikungunya virus, Middelburg virus, Semliki Forest virus and O’nyong-nyong virus) and Flavivirus (Yellow Fever virus, Dengue virus, West Nile virus) genera. Moreover, the sera collection will allow the investigation of the seroprevalence of emerging viruses like SARS-CoV-2 over time. Among ARI and GI patients presenting with a fever, and for which test results are negative for respectively all respiratory and gastrointestinal pathogens, whole blood samples will also be analysed with the *Fever chip*.

A subset of positive specimens (dependent on the pathogens found, e.g. specimens positive for human metapneumoviruses, respiratory syncytial viruses, enteroviruses) will be further sequenced by High Throughput Sequencing (HTS) methodology (amplicon-based enrichment, hybridization capture or shotgun sequencing when relevant) in order to generate full length genomic data for subsequent analyses. Specimens tested positive for SARS-CoV-2 by real time PCR will be sequenced on-site via an amplicon-based full genome sequencing approach [31] using Oxford Nanopore Technology (ONT) devices. Nucleic acid sequences will be provided to the scientific community through respective data repositories.

Most bacteriological and molecular analyses are conducted at national partner laboratories and respective staff are trained extensively (e.g. through multiple workshops and regular follow-up and discussion of data quality) in the use of relevant techniques. Standard operating procedures of laboratory assays are harmonized among participating laboratories. In addition to using standardized assays, reference strains are used in bacteriological analyses and quality control. Protocols for antimicrobial susceptibility testing were developed based on the European Committee on Antimicrobial Susceptibility Testing guidelines [32]. For quality monitoring of molecular testing, a blinded PCR control panel of positive and negative control specimen will be provided to all partner laboratories. For biological specimens, adherence to guidelines for recommended temperature and processing times (i.e., storage of specimens for maximum 2 h at ambient temperature or for maximum 12 h at 4 °C, immediate storage at − 80 °C after aliquoting) were implemented through standard guidelines. In case of irregularities, sentinel sites will enter the details on the concerned specimen in the database and post hoc re-evaluation/repetitions of respective laboratory testing will be conducted. All laboratory results are recorded in respective laboratory results forms.

### Patient follow-up

A follow-up interview will be conducted by trained surveillance staff via telephone to determine disease outcome of patients after 1 month, i.e. patient recovery, ongoing disease symptoms, and subsequent hospital stays. Patients complete a respective one-month follow-up questionnaire.

### Data management and quality control

All data collection and analyses are conducted according to standard operating procedures. All study forms (e.g. case investigation form, one-month follow-up form, sampling form, laboratory results forms) were translated and validated in French for CIV, BF and DRC and English for RSA. Paper data collection forms are completed on site at the point of care and then entered by trained data clerks into a customized database specific for ANDEMIA including a range of plausibility checks to avoid data entry errors and missing data. Visual data validation of data entered is done by a second person and marked as validated. Regular data management reports are sent to sites for validation and correction of data as needed. Regular trainings on the use of the database and data management for ANDEMIA are conducted at the site and country level. All current study form versions are made available to ANDEMIA staff on the database platform.

### Analysis plan

Disease aetiologies and patient characteristics will be descriptively summarized by those with ARI, GI and AFDUC. A latent class analysis integrating the multiple test results among cases only will be conducted to further evaluate aetiology. Among the cases and controls, the distribution of aetiologies will be compared using logistic regression to estimate corresponding odds ratios. Expanding on the odds ratio approach, recent novel approaches using Bayesian estimation including elements of attributable fraction and latent class analyses will be explored in order to better integrate multiple types of measurements, correct for test performance, and accommodate a larger number of pathogens. Patient clinical, demographic, socio-economic and behavioural risk factors will be assessed using conditional logistic regression analysis. Trend analyses such as time series methods will be used to determine if there is a seasonal pattern of certain pathogens, e.g. respiratory viruses. These analyses will be conducted with R and STATA.

A series of pathogen-specific molecular epidemiological and phylogenetic studies will also be conducted for pathogens of global importance, e.g. respiratory viruses including SARS-CoV-2. Here, HTS approaches will be used in order to generate full genome datasets. Genomic HTS data will be analysed in maximum-likelihood as well as Bayesian frameworks [33, 34] in order to infer on divergence times and geographic origins of pathogen lineages. For SARS-CoV-2 sequence generation and analysis, protocols provided by the ARTIC network will be used [35], and virus lineages will be assigned using the Pangolin nomenclature [36].

## Discussion

The ANDEMIA network aims to establish sentinel surveillance of ARI, GI and AFDUC given their high disease burden worldwide. While studies on those diseases exist, the novelty of ANDEMIA lies in its comprehensive diagnostic approach of all three highly interrelated disease syndromes. This way, ANDEMIA expects to address knowledge gaps regarding the aetiologies of those disease syndromes. The data generated here will build on findings from other studies like the Global Approach to Biology Research, Infectious diseases and Epidemics in Low-income countries (GABRIEL) network, the PERCH study on childhood pneumonia aetiology in the Americas, Africa and Asia [37, 38] and the GEMS study on causes of diarrhoea in children in Africa and Asia [12, 39], where similar molecular diagnostic panels were used. However, the inclusion of diverse study sites in different climatic, demographic and biodiversity settings will further allow for the comprehensive investigation of the interplay between circulating pathogens and related factors in humans, including zoonotic pathogens from wildlife and livestock.

ANDEMIA contributes to a better understanding of global infectious disease dynamics and supports the rapid detection and monitoring of outbreaks, as demonstrated by its ability to easily include SARS-CoV-2 in the existing sampling and testing strategy of respiratory specimens. It now offers the possibility to investigate SARS-CoV-2 dynamics in the past months and prospectively. This illustrates the potential and flexibility of such long-term disease surveillance studies. The study also reinforces routine laboratory testing to build diagnostic capacity of treatable disease at all hospital sites, such as bacterial culture methods and AMR testing. Furthermore, it fosters South-South collaboration among individuals and institutions in the selected Francophone and Anglophone African countries, which also leads to a more sustainable impact. ANDEMIA intends to use the surveillance data to develop targeted interventions, assess their effectiveness and consider other in-depth investigation studies. Data will be fed back into national health information systems to facilitate translation of findings into practice.

## Data Availability

Not applicable.

## Abbreviations

AFDUC: Acute febrile disease of unknown cause
AMR: Antimicrobial resistance
ANDEMIA: African network for improved diagnostics, epidemiology and management of common infectious agents
ARI: Acute respiratory infection
BF: Burkina Faso
CHR: Centre hospitalier regional
CHU: Centre hospitalier et universitaire
CIV: Côte d’Ivoire
COVID-19: Novel coronavirus disease 2019
CSF: Cerebrospinal fluid
DRC: Democratic Republic of the Congo
EDTA: Ethylenediaminetetraacetic acid
FIEBRE: Febrile illness evaluation in a broad range of endemicities
GABRIEL: Global approach to biology research, infectious diseases and epidemics in low-income countries
GEMS: Global enteric multicenter study
GI: Acute gastrointestinal infection
HTS: High throughput sequencing
INRB: Institut National de la Recherche Biomédicale
LANADA: Laboratoire National d’Appui au Développement Agricole / Laboratoire Central dePathologie Animale
NICD: National Institute for Communicable Diseases
PCR: Polymerase chain reaction
PERCH: Pneumonia etiology research for child health
RKI: Robert Koch-Institut
RSA: Republic of South Africa
SARS-CoV-2: Severe acute respiratory syndrome coronavirus 2
SSA: Sub-Saharan Africa
UTM: Universal transport media
